# Reply: The performance of the risk of ovarian malignancy algorithm

**DOI:** 10.1038/bjc.2011.225

**Published:** 2011-06-28

**Authors:** T Van Gorp, D Timmerman, I Vergote

**Affiliations:** 1 Division of Gynaecological Oncology, Department of Obstetrics and Gynaecology, Universitair Ziekenhuis Leuven, Katholieke Universiteit Leuven, Herestraat 49, 3000 Leuven, Belgium; 2 Division of Gynaecological Oncology, Department of Obstetrics and Gynaecology, MUMC+, GROW – School for Oncology and Developmental Biology, PO Box 5800, 6202AZ Maastricht, The Netherlands

**Sir,**

We appreciate the data and comments provided by Langmár *et al* (2011) regarding our recent report ‘HE4 and CA125 as a diagnostic test in ovarian cancer: prospective validation of the Risk of Ovarian Malignancy Algorithm’ (Van Gorp *et al*, 2011).

In their letter Langmár *et al* suggest that we did not start this prospective study as a generic pelvic mass diagnostic study. They draw this conclusion by the fact that we started this study in 2005, and yet the studies from Moore *et al* were only published in 2008 and 2009 (Moore *et al*, 2008, 2009). However, we must point out that several publications on HE4 were already published between 2003 and 2005 (Hellstrom *et al*, 2003; Lu *et al*, 2004; Drapkin *et al*, 2005; Rosen *et al*, 2005). Between August 2005 and March 2009, 432 consecutive patients with a pelvic mass, who planned to undergo an operation, were eligible to participate in our study. Five patients withdrew consent during the study. Seventeen patients did not have the serum sample taken, or the volume of the retrieved serum was insufficient to perform the marker assays. Pathology reports were missing in 21 patients because the patients were eventually not operated upon due to poor prognosis or patients’ decline, or because there was no biopsy taken and therefore no proven histological diagnosis. The remaining 389 patients participated in this study. In 2008 we were confronted with the results from Moore and colleagues. Since they published a significant difference between the performance of CA125 and CA125+HE4 in a study with only 233 patients, one could expect that we should have been able to show a significantly different result with our 389 patients, which is the largest monocentre cohort study on HE4. In fact, if one uses the AUCs of CA125 (0.836) and the combination of CA125 and HE4 (0.914) from the article of Moore *et al*, with an alpha level of 0.05 and a beta level of 0.20, one can obtain a required minimum sample size of 86 in each group. With a sample size of 389 patients, we broadly exceeded the required number of samples.

The authors also suggest that there was a selection bias, with an increased amount of cancers, postmenopausal patients, mucinous tumours, borderline tumours and metastatic tumours of extra-ovarian origin. Indeed, the bigger proportion of these specific categories is a reflection of a patient population with adnexal masses in a tertiary centre as ours. Recruitment was also largely done in collaboration with our ultrasound department, which is an expert centre in the diagnosis of pelvic masses (Timmerman *et al*, 2010). Hence, there was a relatively large proportion of stage I and borderline tumours in our study, which are more difficult to differentiate from their benign counterparts compared with stage III or IV ovarian cancers. The fact that we were not able to show a statistical difference between CA125 and ROMA illustrates that the ROMA algorithm does not seem to be of any benefit in this particular setting, which in our opinion is an important finding for many centres. Moreover, in this setting, with more difficult tumours to diagnose, the addition of a good diagnostic tumour marker could have been of great importance, and yet, the addition of HE4 to CA125 does not seem to fulfill these expectations.

Langmár *et al* also claim that the non-significant differences between the AUCs of the different tests cannot exclude a gain in diagnostic accuracy with statistical certainty. This strikes us as a *contradictio in terminis*. As mentioned in the methods section, we used the non-parametric method as described by DeLong *et al* (1988) to calculate differences in AUCs. The method of DeLong *et al* is the most widely used method to perform these comparisons. We do not dispute the calculated differences in AUCs and 95% confidence intervals by Langmár *et al*, but as the *P*-level was 0.17 we were not able to reject the null hypothesis, and thus in our setting this means that the results were not statistically different. All comparisons and corresponding *P*-values are clearly mentioned in our results section and are illustrated in the tables and figures.

Finally, Langmár *et al* question our interpretation of the HE4 threshold. They state that the optimal threshold depends on the characteristics of the population and the consequences of the true and false test outcomes. Again, we fully agree with this last remark. In the diagnosis of ovarian cancer one should try to detect as many of the ovarian cancers as possible, and yet avoid unnecessary surgeries for benign cysts. This implies that sensitivity is very important; however, increasing morbidity, costs, pain and fear by performing midline laparotomies for benign cysts are also unacceptable. Therefore we have looked at the optimal cut-off rather than a cut-off at a fixed sensitivity or specificity. As mentioned in our article, the optimal cut-off corresponds to the point on the ROC curve with the highest accuracy. As we did not use healthy controls, we did not intend to find a cut-off for HE4 that could be used in screening. Moreover, we clearly stated that using a cut-off of 150 PM, as suggested in the product insert, would result in high specificity (96.5%), but in very low sensitivity (50.3%). Therefore, a cut-off of 150 PM cannot be used in a diagnostic situation in a population as ours. A cut-off of 70 PM is more appropriate and very close to our calculated optimal cut-off of 72.2 PM.

In conclusion, we understand that these negative results for this promising HE4 marker and ROMA algorithm are disappointing. We can only conclude that neither HE4 nor the combination of HE4 and CA125 in the ROMA algorithm seems to be an improvement in the diagnosis of ovarian cancer in patients with an adnexal mass as referred to a tertiary centre as ours.
